# The Impact of Laser Energy Levels on In Situ Fenestrations of Aortic Stent Grafts: An On-Bench Study

**DOI:** 10.3390/bioengineering13091085

**Published:** 2026-09-19

**Authors:** Teodora Ormandzhieva, Florian Elger, Marcello Silvano, Bernd Niemann, Eberhard Grambow

**Affiliations:** 1 Department of Cardiac, Thoracic and Vascular Surgery, University Medical Center Goettingen, 37075 Göttingen, Germanyeberhard.grambow@med.uni-goettingen.de (E.G.); 2 Department of Vascular Medicine (Vascular and Endovascular Surgery), DRK Kliniken Berlin, 13359 Berlin, Germany; 3 Division of Vascular and Endovascular Surgery, Department of Cardiac, Thoracic, Vascular Sciences and Public Health, University of Padua, 35122 Padova, Italy; 4 Department of General, Visceral, Thoracic, Vascular and Transplantation Surgery, University Medical Center Rostock, 18057 Rostock, Germany

**Keywords:** laser in situ fenestration, aortic stent graft, excimer laser, fenestration protocol, L-ISF, bulging, fraying

## Abstract

(1) Background: Laser in situ fenestration (L-ISF) is an endovascular technique used to preserve target vessel patency in urgent or emergency endovascular aortic repair when fenestrated/branched solutions are unavailable or unsuitable, or as a bailout technique for fenestration or branch misalignment. This on-bench study compared different laser energy levels in five commercially available endografts to assess fabric damage and evaluate fenestration quality, dimensions, and recoil. (2) Methods: A total of 102 fenestrations were created using a 2.0 mm excimer laser catheter at low (45 mJ/mm^2^) or high (60 mJ/mm^2^) energy levels and dilated with a 6 mm plain balloon in five polyester endografts (Zenith Alpha, RelayPro, Endurant IIs, Valiant Captivia, Hercules). High-magnification microscopy assessed morphology and fabric damage after puncture, after dilation, and after 24 h. Quantitative measurements included diameters, area, and recoil under standardized study conditions. (3) Results: Valiant exhibited clearer puncture margins and no bulging; Hercules showed frequent tearing and more bulging and warp-aligned recoil at high energy. In Endurant IIs, low-energy L-ISF produced larger diameters and area but also greater recoil. RelayPro revealed stronger surface recoil with high energy. (4) Conclusion: Both low- and high-energy L-ISF were feasible across all tested grafts. A low-energy approach may be preferable for Endurant IIs, potentially reducing the risk of bridging stent graft stenosis.

## 1. Introduction

Complex aortic repair for juxta-/pararenal or thoracoabdominal aortic aneurysms typically involves the use of custom-made or off-the-shelf fenestrated and branched stent grafts.

Laser in situ fenestration (L-ISF) represents an alternative option to maintain the patency of target vessels (TVs) when standard options are unfeasible due to excessively long manufacturing times and/or unsuitable anatomies.

Several studies have evaluated the technique in vivo with encouraging results, and in vitro analyses have attempted to identify the most effective combination of puncture and fenestration dilation strategies [1,2].

Currently, two main laser modalities are used for L-ISF: diode and excimer laser systems [3]. Diode laser-assisted fenestration is more widely accessible and cost-effective but relies on thermal energy delivery, which may result in increased thermal injury to the vessel wall and graft material. In contrast, excimer laser systems use photochemical energy, enabling more precise ablation with reduced thermal spread, albeit at higher cost and greater technical complexity [4].

Previous studies reported satisfactory fenestration quality and dimensions using larger-caliber laser catheters (2.0–2.3 mm), followed by plain balloon dilation (PBD) with 6–8 mm balloons [5,6]. However, higher laser energy settings may lead to excessive graft disruption and potential vessel injury [7], and there is currently no clear consensus regarding the optimal energy parameters for L-ISF.

The present study aimed to evaluate and compare the in vitro performance of low- versus high-energy excimer laser-assisted fenestration across a range of commercially available stent grafts, with the goal of identifying device-specific optimal energy settings.

## 2. Materials and Methods

### 2.1. Fenestration Creation

A total of 102 L-ISFs were obtained in five different commercially available polyester endografts (n = 1 specimen per stent-graft model):

- Zenith Alpha (Cook Medical, Bloomington, IN, USA);
- RelayPro (Terumo Aortic, Sunrise, FL, USA);
- Endurant IIs (Medtronic Vascular, Santa Rosa, CA, USA);
- Valiant Captivia (Medtronic Vascular, Santa Rosa, CA, USA);
- Hercules (Lombard Medical Endovastec, Oxfordshire, UK).

All fenestrations were performed using a 308 nm excimer laser system and a 2.0 mm over-the-wire (OTW) laser atherectomy catheter (Spectranetics Corporation, Colorado Springs, USA). Two different laser setting schemes were used: in group A (low energy), the laser was set to a fluency of 45 mJ/mm^2^ and a frequency of 25 Hz, while in group B (high energy), the setting was 60 mJ/mm^2^ of fluency and 25 Hz of frequency. Both schemes were considered based on the available literature [6].

In all cases, the graft fabric was submerged in a 37 °C isotonic 0.9% saline bath to mimic the in vivo situation, and the laser catheter was positioned at 90° to the graft surface. A short laser burst was delivered while gently advancing the catheter through the polyester layers.

After fabric puncture, the fenestration was dilated with a 6 mm balloon (Admiral Xtreme™, Medtronic, Minneapolis, MN, USA), as is typically done in our clinical experience with larger (2.0 and 2.3 mm) laser fibers [8].

One physical specimen of each stent-graft model was used. Multiple spatially distinct fenestrations were created within each specimen using either the low- or high-energy protocol. Accordingly, the individual fenestrations represent technical within-specimen replicates rather than independent graft-level replicates. All fenestrations were performed by the same operator team.

Overall, the number of fenestrations per graft was the following:

- Zenith Alpha n = 24 (group A = 12, group B = 12);
- RelayPro: n = 24 (group A = 12, group B = 12);
- Endurant IIs n = 20 (group A = 10, group B = 10);
- Valiant Captivia n = 14 (group A = 7, group B = 7);
- Hercules n = 20 (group A = 10, group B = 10).

### 2.2. Fenestration Assessment and Outcomes

Fenestrations were evaluated using high-magnification microscopy (×140; DinoLite AM8917MZTL and DinoCapture 2.0, Dunwell Tech., Torrance, CA, USA). Standardized microscopic images were acquired after initial laser puncture, immediately after balloon dilatation, and 24 h after dilatation. Following balloon dilatation, each fenestration was assessed for morphology and fabric damage, including shape, tearing, fraying, shredding, and bulging according to the predefined criteria described below. Quantitative assessment included measurement of the maximum diameters aligned with the warp and weft directions of the fabric and determination of the fenestration surface area. The same dimensional parameters were reassessed after 24 h to quantify early post-dilatation fabric recoil.

The following parameters were assessed:

- Fenestration shape, including elliptical, round, slit-like, and square;
- Fenestration technical features, including the presence of tearing, fraying, shredding, and bulging;
- Fenestration weft-aligned and warp-aligned diameters, and fenestration area;
- Fenestration weft-aligned and warp-aligned diameters and area recoil (relative reduction in the fenestration dimensions) 24 h after dilatation.

**Figure 1:**
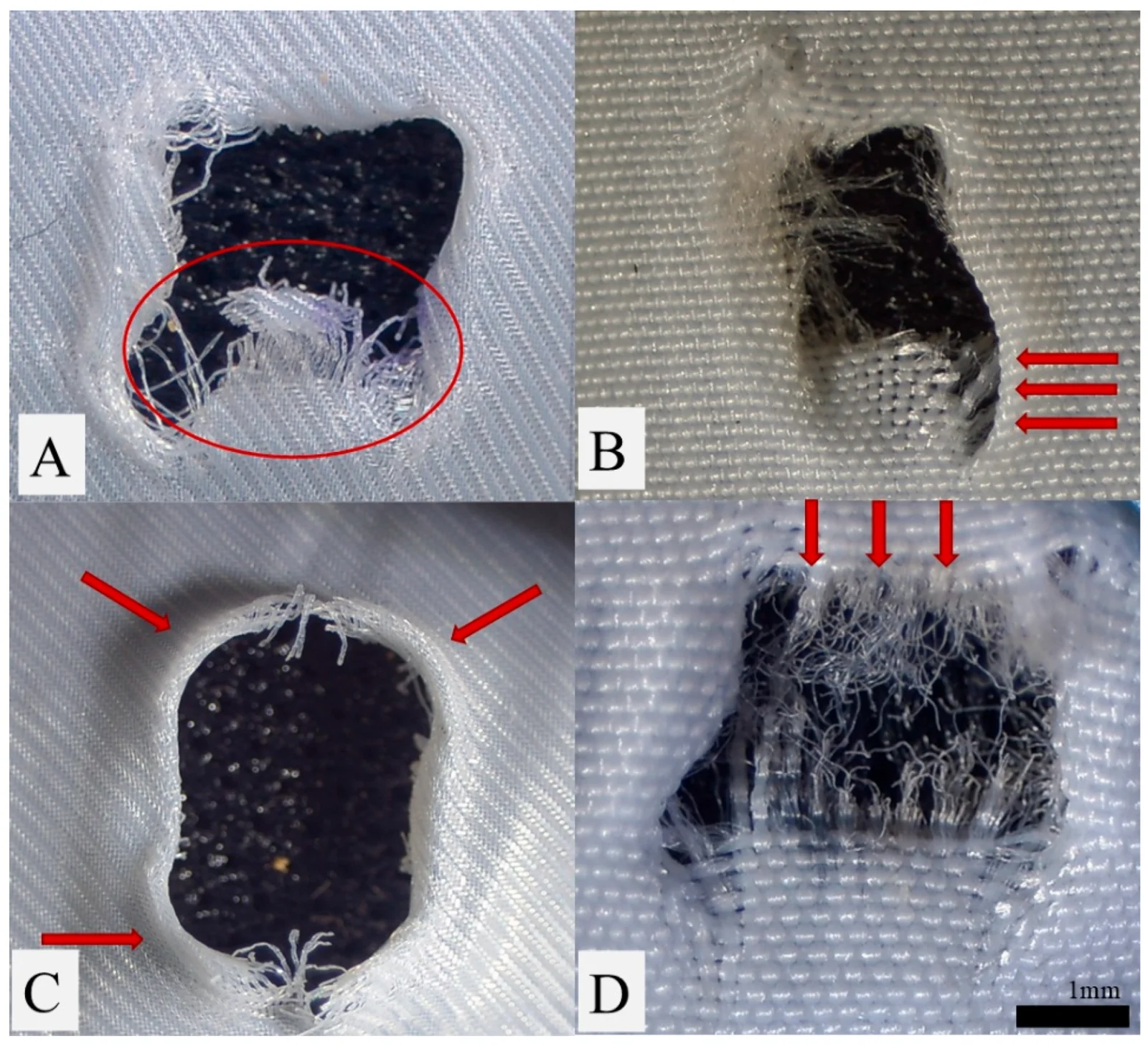
Exemplary images of fenestration features after laser fenestration and balloon dilation: (**A**) Shredding in the Valiant Captivia, (**B**) Tearing in the Zenith Alpha, (**C**) Bulging in the Hercules, (**D**) Fraying in the Endurant IIs.

The following definitions were applied to describe the fenestration morphology:

Fabric tearing was defined as a visible fabric tear (>1 mm) beginning from the fenestration edge, occurring in either the weft or warp direction. Fraying was defined as the occurrence of extensive (>5) disrupted fibers at the fenestration edge. Shredding was defined as the presence of larger fragments of graft material partially (>⅓ of the fenestration surface) obstructing the fenestration opening. Bulging was defined as an outward deformation of graft fabric around the fenestration without fiber disruption (Figure 1).

Fenestration recoil was quantified as the absolute and relative difference between the initial and 24 h measurements (for both surfaces and diameters) and expressed as number and percentage respectively. The time between the measurements was chosen to ensure comparability with previous studies [8,9]. All features and measurements were analyzed by two independent observers.

The primary outcome of this study was the presence of L-ISF-associated fenestration damage, including fabric tearing and shredding.

Secondary outcomes included fenestration dimensions (surface, warp- and weft-aligned lengths), additional fenestration features (fenestration bulging and fraying) and 24 h recoil after high- and low-energy L-ISF.

### 2.3. Statistical Analysis

Statistical analyses were performed using SPSS Statistics for Windows (IBM, version 24; Chicago, IL, USA). The distribution of continuous variables was assessed using the Shapiro–Wilk test. Continuous variables are presented as median and interquartile range (Q1–Q3), and categorical variables as absolute numbers and percentages.

Each endograft type was analyzed separately. Within each investigated endograft specimen, multiple spatially distinct fenestrations were created under low- and high-energy conditions. Statistical comparisons were restricted to fenestrations created with low versus high energy within the same endograft type; no comparisons were performed across different endograft types. Continuous variables were compared using the independent-samples t-test when normally distributed and the Mann–Whitney U test otherwise, whereas categorical variables were compared using Fisher’s exact test.

Given the technical-replicate structure of the experiment and the absence of independent graft-level replication, these statistical comparisons were considered exploratory and hypothesis-generating. Nominal *p* values should therefore be interpreted as descriptive indicators of within-specimen differences rather than as confirmatory population-level inference for the respective stent-graft platforms. To account for the number of comparisons across multiple outcome parameters, a two-sided *p* value < 0.05 was considered statistically significant.

Interobserver reliability for quantitative fenestration measurements was assessed using a two-way mixed-effects intraclass correlation coefficient (ICC) model for absolute agreement based on single measurements. ICCs were calculated for warp- and weft-aligned fenestration diameters and fenestration area immediately after balloon dilation and at 24 h follow-up. ICC values > 0.90 were considered indicative of excellent interobserver reliability (Table 1).

## 3. Results

### 3.1. Fenestration Quality Assessment

**Figure 2:**
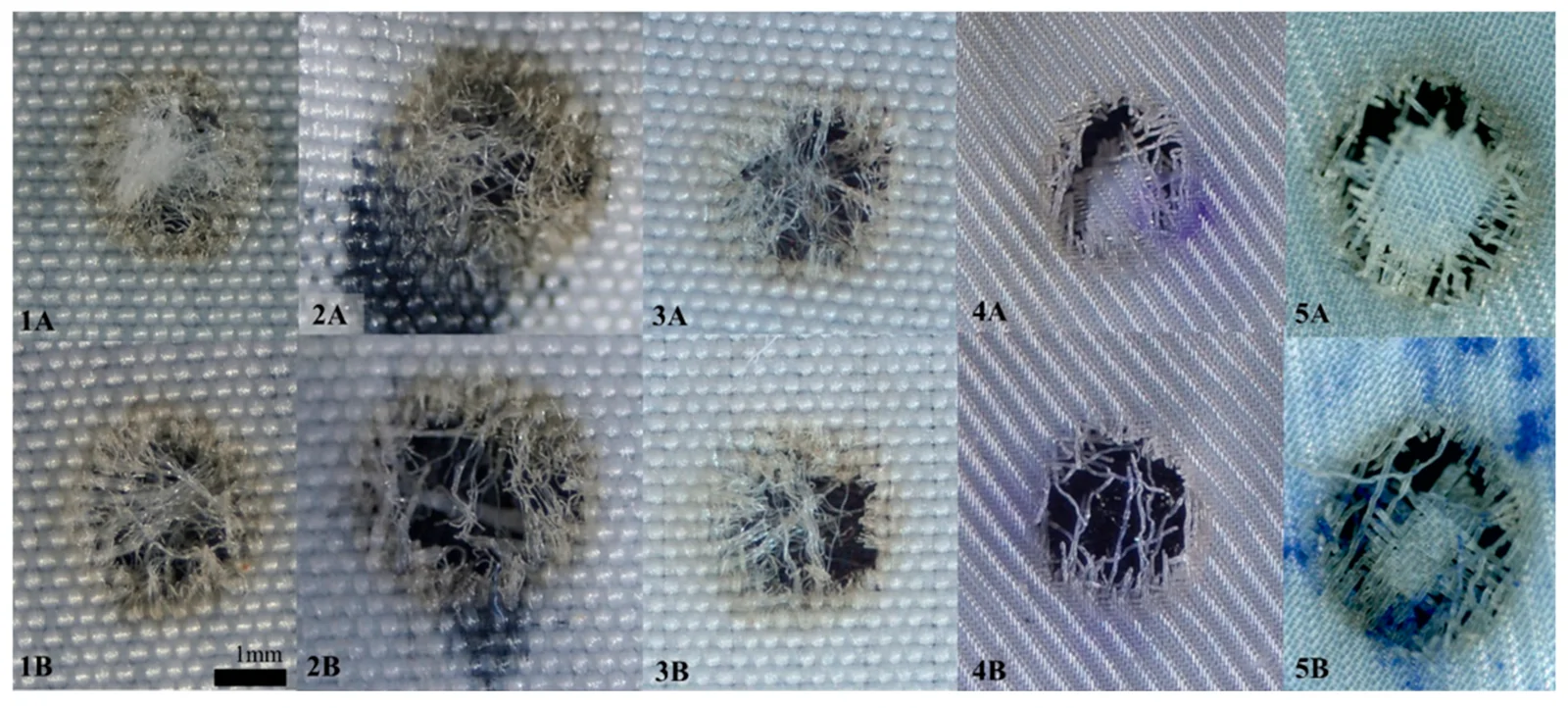
Effect of low (**A**) and high (**B**) energy levels on laser fenestrations: (**1A**,**1B**) Zenith Alpha, (**2A**,**2B**) Endurant IIs, (**3A**,**3B**)—RelayPro, (**4A**,**4B**)—Valiant Captivia, (**5A**,**5B**)—Hercules (pictures taken prior to PTA, blue and black spots were left over from fenestration marking prior to ISF).

Following the initial laser puncture, the quality of the fenestrations was similar in both groups. The fenestration hole was frequently surrounded or covered by disorganized melted fibers or fabric shreds. A brown burned halo was visible around the fenestrations. High-energy fenestrations tended to be more widely patent, with fewer fibers or smaller fragments obstructing the orifice (Figure 2). All the multifilament grafts (RelayPro, Zenith Alpha, Endurant IIs and Hercules) showed a significantly denser fiber web after the initial puncture, compared to the monofilament fabric (Valiant), but obvious fabric shreds were only visible in the Hercules and in the Valiant in group A (low energy). Additionally, laser fenestration in the Valiant graft was characterized by sharper edges with fewer peripheral melted fibers compared to the other investigated grafts under both high- and low-energy settings.

Following laser fenestration, the balloon passage was successfully achieved through all fenestrations with no technical difficulty, and plain balloon dilatation was successfully performed on all fenestrations.

After plain balloon dilatation, all grafts, in both groups, primarily displayed elliptical and round fenestration shapes, with the sole exception of Endurant IIs, presenting with only square fenestrations in group B (high energy) and mostly elliptical fenestrations in group A (low energy) (*p* = 0.003).

**Figure 3:**
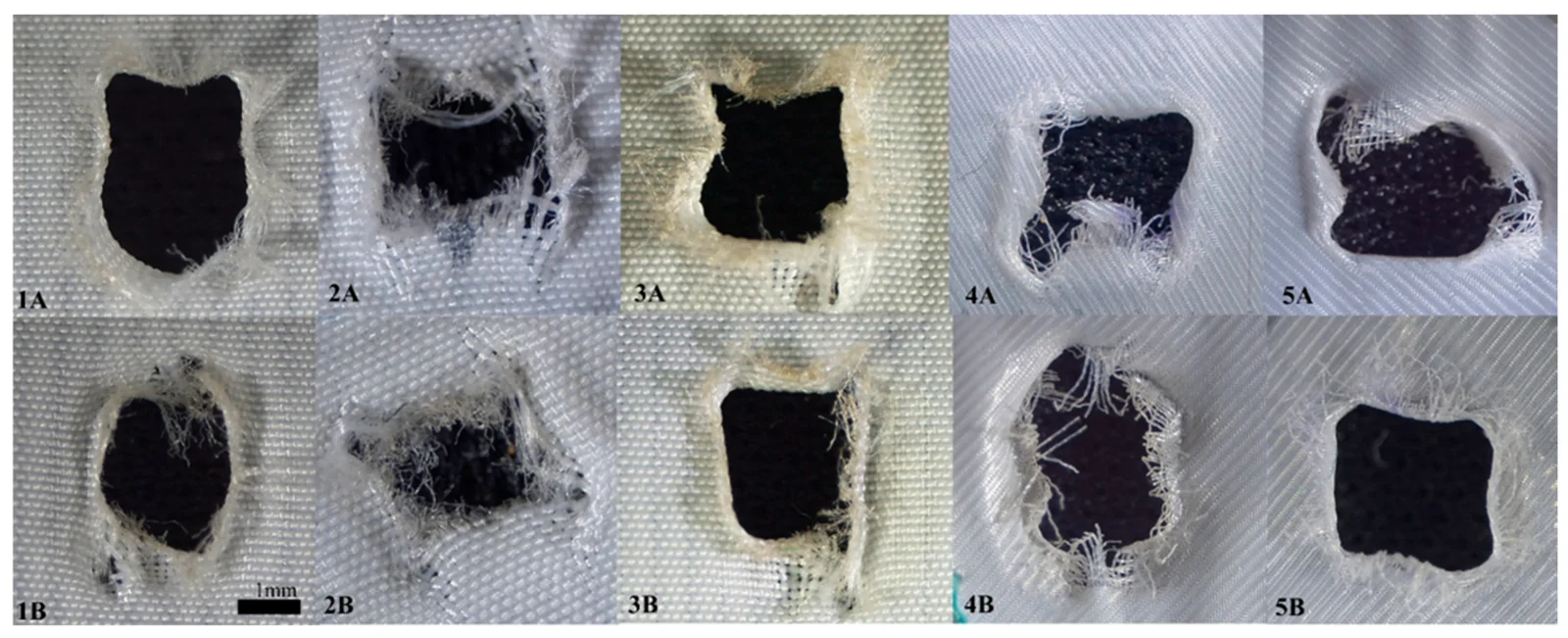
Changes in the appearance of the laser fenestrations after PTA—low (**A**) energy level and high (**B**) energy level: (**1A**,**1B**) Zenith Alpha, (**2A**,**2B**) Endurant IIs, (**3A**,**3B**) RelayPro, (**4A**,**4B**) Valiant Captivia, (**5A**,**5B**) Hercules (images taken at 140× magnification).

Fabric tearing was absent or modestly present in all fabrics in both groups, without any significant difference. Only the Hercules graft exhibited a high incidence of tearing (8, 80%) in both groups (Table 2). Interestingly, the only monofilament graft in this study, the Valiant, showed very limited signs of tearing in either group. Shredding was more prevalent in the Endurant IIs graft than in the others (7, 70%), again, with no differences between the two groups. Fraying was diffusely present in all multifilament grafts (less so in the Valiant, particularly in group B (high energy)), regardless of the fenestration protocol. The only significant difference in bulging was identified in the Hercules graft in group B (high energy) (3 vs. 10, *p* < 0.001) (Figure 3).

Morphological and quantitative characteristics of laser in situ fenestrations according to stent-graft type and laser-energy setting. Data are presented as median [Q1–Q3] or n (%), as appropriate. Low- versus high-energy comparisons were performed separately within each stent-graft specimen. Individual fenestrations represent technical within-specimen replicates, as only one physical specimen of each stent-graft model was investigated. All *p* values are nominal, unadjusted, and reported for exploratory purposes only; they should not be interpreted as confirmatory population-level inference.

### 3.2. Fenestration Dimensions

Fenestration diameters and surfaces were generally comparable between the two groups for all grafts. However, in the Endurant IIs graft, the low-energy fenestration protocol (group A) seemed to allow larger weft-aligned diameters (2.8 mm vs. 2.5 mm, *p* = 0.027) and especially wider fenestration areas (7.8 mm^2^ vs. 6.5 mm^2^, *p* = 0.007) compared to the high-energy fenestration protocol. Additionally, fenestration dimensions and surfaces in this graft were inferior in both groups when compared to other grafts. In the other examined grafts, fenestration dimensions appeared generally larger in group B (high energy), even if not significantly. Only the fenestrations in the RelayPro graft demonstrated a slight superiority in warp-aligned diameter (*p* = 0.020) in group A (low energy) (Table 2).

### 3.3. Fabric Recoil

A more prominent recoil along the warp-aligned axis was observed in the Endurant IIs graft in group A (low energy) (16.4% vs. 11.3%, *p* = 0.031) and in the Hercules graft in group B (high energy) (9.3% vs. 16.7%, *p* = 0.022). The RelayPro was the only graft to demonstrate a significant difference in surface recoil (25.1% in group A (low energy) vs. 33.6% in group B (high energy); *p* = 0.026), although no corresponding difference in final surface area was observed (*p* = 0.178).

## 4. Discussion

This paper is a pilot in vitro proof-of-concept analysis designed to assess dimensions, morphological features and fabric damage of fenestrations after low- and high-energy L-ISF in commercially available stent grafts.

Three key open questions relating to the role of laser energy level may be identified: (1) whether fenestration dimensions depend on energy settings, (2) whether different energy levels correlate with the extent of fabric damage (e.g., tearing or shredding), and (3) the potential effect of laser energy on the vessel wall [3]. Given the study’s in vitro setting, this paper focuses on the first two aspects.

Our preliminary results suggest a general and broad comparability between the two L-ISF energy settings, especially in terms of fabric damage. Limited, but possibly significant, differences were observed between the two fenestration patterns in each graft, especially in Endurant IIs, which shows high fabric recoil and a polyester structure requiring extensive dilatation effort [10,11]. In this graft a low-energy fenestration scheme yielded wider fenestrations that might better accommodate a bridging stent graft (BSG).

### 4.1. Fenestration Features

In this study, the initial fenestration morphology was similar to that previously described by Lin et al. [5]. Fabric tearing occurred more frequently in the Hercules graft and did not seem to depend on the applied laser energy level.

The clinical relevance of fabric tearing remains uncertain. Some authors suggest that such disruptions may compromise fenestration stability [5,12]. Conversely, other investigations indicate that limited tearing may be incorporated into the fenestration following deployment of a bridging stent graft without structural deterioration, even after prolonged mechanical fatigue [9,12].

In contrast, fabric shredding and fraying are considered more likely to carry an embolic risk. It has been proposed that small, loose, free-floating fragments may detach from fenestration edges during subsequent stenting procedures [5,12]. Sonesson et al. [13] specifically investigated this phenomenon but did not detect any measurable particle release. In the present experience, the incidence of this feature did not clearly correlate with the laser energy level, although the Endurant IIs graft seemed to be more prone to shredding after pre-dilation.

Bulging likely reflects the elastic behavior of the graft fabric as it deflects around the zone of applied force. In this work, this occurrence was widely observed exclusively after plain balloon dilatation. Previous studies suggest that cutting balloons may mitigate bulging by producing small rim cuts or larger tears [9]. While bulged edges could theoretically improve sealing after bridging stent graft deployment, supporting evidence is currently lacking, and their clinical significance remains unclear at this point.

### 4.2. Fenestration Dimensions

Small fenestrations are generally considered a risk factor for bridging stent graft stenosis [5,12]. A standard 6 mm diameter bridging stent graft has a surface area of approximately 28 mm^2^, which increases when flared, whereas most L-ISFs are significantly smaller, even after pre-dilation.

Although larger balloons can be used to achieve more comparable dimensions, these have been associated with unpredictable fabric damage [5,6]. However, previous experience with smaller (6 mm) balloons showed favorable outcomes, with low rates of in-stent stenosis [9].

Lin et al. tested different stent grafts, including the Endurant IIs, applying similar energy levels to those employed in our study (low: 30 mJ/mm^2^; high: 60 mJ/mm^2^), obtaining comparable fenestration dimensions [5,12]. However, the present findings suggest that the low-energy protocol (group A) provided wider fenestrations in the Endurant IIs, despite greater 24 h recoil.

Grima et al. also applied different energy levels and dilated the fenestrations with an 8 mm balloon [6]. No tearing was found in the Zenith Alpha at either energy level, whereas tearing was identified in some cases in our study. However, no information was provided regarding the fenestration dimensions.

These in vitro observations are supported by early in vivo clinical data demonstrating that in situ laser fenestration is a feasible and reliable bailout strategy in emergency settings. Some authors reported midterm outcomes following antegrade L-ISF performed with a 0.9 mm excimer laser fiber and subsequent percutaneous transluminal angioplasty using non-compliant and cutting balloons [3]. Owing to the smaller laser fiber diameter and balloon size, direct comparison with ISF strategies employing larger laser fibers is not yet possible. Nevertheless, the study demonstrated good procedural feasibility and a midterm target vessel patency rate of 97% in an in vivo setting [14].

Although endoleaks and re-interventions were reported, neither study included a systematic assessment of fabric damage at the fenestration site, and no direct association between laser-induced fabric injury and endoleak occurrence was investigated.

### 4.3. General Considerations on L-ISF Techniques

L-ISF encompasses a wide range of technical approaches, reflecting substantial variability in clinical practice. Beyond excimer laser systems, diode laser-based fenestration represents an alternative modality, characterized by thermal rather than photochemical energy delivery. This difference may affect graft–laser interaction, particularly in terms of thermal injury, edge morphology, and material behavior, and warrants further investigation [4].

Additional variability arises from the choice of laser fiber diameter, catheter design, and adjunctive techniques, including the use of cutting versus non-compliant balloons, sequential dilation strategies, or alternative crossing methods. Smaller laser fibers may provide greater control but require more extensive post-dilation, whereas larger-caliber systems may achieve wider initial fenestrations at the cost of increased material stress.

From a technical perspective, the interaction between laser energy and graft fabric is complex and depends on polymer composition, yarn structure, and weave orientation. Thermal and photochemical effects may alter fiber integrity, induce localized melting or disruption, and modify mechanical properties such as elasticity and creep behavior [15,16,17].

Thermal-mechanical findings from excimer (308 nm) ISF bench testing should be considered in the context of the experimental environment, as the 37 °C isotone, 0.9% saline bath enhances convective heat dissipation and may reduce the extent and duration of thermal injury compared with air. At the same time, excimer ablation in fluid can generate vapor bubbles that affect local energy coupling and introduce variability in fenestration edge morphology, particularly when balloon dilation is performed immediately after L-ISF [18,19].

Laser energy likely induces localized alterations in polymer structure and fiber elasticity that are not fully captured by light microscopy imaging alone. While scanning electron microscopy studies by Lin et al. demonstrate fraying or tearing at fenestration margins without consistent macroscopic melting, other reports suggest that laser-induced thermal effects may lead to localized burning or melting, disrupting yarn integrity and facilitating tear propagation [12,20]. These changes may modify the mechanical properties of the graft, including reduced elastic recoil due to thermal softening followed by re-hardening upon cooling.

Beyond these technical factors, spatial optimization of the fenestration and bridging stent graft configuration represents an important consideration in clinical practice, particularly in complex and patient-specific aortic anatomies. This aspect was not reproduced in the present bench model, which was deliberately designed to isolate the interaction between laser energy and graft fabric. Future experimental models also accounting for patient-specific anatomy and spatial graft configuration may provide a more comprehensive assessment of L-ISF performance under clinically representative conditions.

Subsequent balloon dilation may further exacerbate creep and permanent deformation in the thermally affected regions. The relative contributions of laser energy, graft material properties, and mechanical loading, however, remain difficult to disentangle. Depending on the fabric type, the present study may therefore provide initial insights into early post-ISF fabric behavior, both immediately after fenestration and within the first 24 h.

These considerations underscore the need for standardized in vitro testing protocols and material-specific evaluation when extrapolating L-ISF performance to clinical practice.

### 4.4. Fabric Recoil

The phenomenon of fabric recoil is obviously present but has not yet been well described or associated with specific clinical outcomes. It can be hypothesized that excessive recoil momentum could cause BSG stenosis [6], while insufficient recoil could compromise the fenestration-bridging stent graft complex in the long term, leading to endoleaks [21]. Conversely, an acceptable magnitude of recoil may serve as a stabilizer. In our study, limited/moderate recoil was observed with the Hercules and the Valiant (both <15%), whereas higher values were found with the Zenith Alpha, the Endurant IIs and the RelayPro. However, the RelayPro graft alone displayed a significant difference in surface area between high- and low-energy fenestration schemes.

### 4.5. Limitations

This study is a preliminary in vitro proof-of-concept analysis. Since no funding was obtained, the number of samples and graft types studied was limited, and no bridging stent grafts were implanted due to high material cost. Therefore, the statistical power of the study remains limited. Additionally, no mechanical testing was performed to evaluate the fenestration durability or fatigue resistance. Such analyses would be essential to assess the long-term behavior of the fenestration-bridging stent graft complex and to explore the impact of different pre-dilation schemes including multiple dilations, larger balloons or cutting balloons.

Comparisons were limited to low-energy (group A) and high-energy (group B) conditions within the same graft, without assessment of differences across graft types.

In this study only excimer laser-based fenestrations were investigated. Other laser fenestration techniques, including diode laser-assisted fenestration, were not evaluated and may show different results given their distinct energy delivery characteristics. Future studies should address these modalities to allow a more comprehensive comparison of laser-based fenestration strategies.

Although the 37 °C isotone saline environment provided standardized conditions approximating physiological temperature and fluid exposure, the present bench model does not reproduce the full anatomical and physiological complexity of in vivo L-ISF. In particular, aortic wall apposition, calcification, pulsatile pressure and flow, vessel angulation, and interactions with surrounding tissues were not represented. These factors may influence laser–graft interaction and fenestration behavior and should therefore be incorporated into more advanced experimental models.

## 5. Conclusions

The present exploratory proof-of-concept analysis suggests that increasing laser energy does not improve fenestration quality, with high- and low-energy settings resulting in comparable degrees of fabric tearing and disruption. Higher energy levels did not consistently translate into larger or more favorable fenestration dimensions; conversely, slightly larger fenestrations were observed with the low-energy protocol in Endurant IIs, highlighting potential endograft-specific differences in fabric response.

Taken together, these findings support further investigation of endograft-specific energy settings and suggest that the lowest energy level that reliably achieves graft penetration may represent a reasonable starting point for future protocols. From a clinical perspective, minimizing laser energy could theoretically reduce unintended injury to the adjacent aortic wall and surrounding tissues, while adequate fenestration dimensions may potentially facilitate bridging stent graft expansion and reduce the risk of stenosis. However, these potential clinical implications were not assessed in the present study and should therefore be interpreted cautiously.

Accordingly, these material-level findings should not be directly extrapolated to clinical practice. Further studies incorporating larger numbers of independent endograft specimens, different bridging stent grafts, mechanical and long-term fatigue testing, and more physiologically representative models are required to determine the clinical relevance and durability of the observed fenestration characteristics.

## Figures and Tables

**Table 1:** Intraclass correlation coefficients for microscopically assessed parameters.

Parameter	ICC	95% CI	Interpretation
Post-dilation horizontal diameter	0.993	0.991–0.995	Excellent
Post-dilation vertical diameter	0.993	0.988–0.995	Excellent
Post-dilation fenestration area	0.997	0.992–0.998	Excellent
Horizontal diameter after 24 h	0.999	0.995–0.999	Excellent
Vertical diameter after 24 h	0.998	0.990–0.998	Excellent
Area after 24 h	0.996	0.993–0.997	Excellent

**Table 2:** Combined Fenestration Analysis—all devices.

		Zenith Alpha			Endurant IIs			Relay Pro			Valiant Captivia			Hercules		
		Group A (N = 12)	Group B (N = 12)	*p*	Group A (N = 10)	Group B (N = 10)	*p*	Group A (N = 12)	Group B (N = 12)	*p*	Group A (N = 7)	Group B (N = 7)	*p*	Group A (N = 10)	Group B (N = 10)	*p*
**Fenestration features**	Tearing	1 (8.3)	3 (25.0)	*0.273*	0	0	–	3 (25.0)	3 (25.0)	*1.0*	1 (14.3)	0	*0.229*	8 (80)	8 (80)	*0.696*
	Fraying	6 (50.0)	8 (66.7)	*0.408*	7 (70)	10 (100)	*0.060*	8 (66.7)	11 (91.7)	*0.132*	4 (57.1)	1 (14.3)	*0.192*	6 (60)	9 (90)	*0.269*
	Shredding	3 (25.0)	3 (25.0)	*1.0*	7 (70)	7 (70)	*1.0*	1 (8.3)	5 (41.7)	*0.590*	6 (85.7)	2 (28.6)	***0.031***	4 (40)	6 (60)	*0.505*
	Bulging	7 (58.3)	10 (83.3)	*0.178*	10 (100)	10 (100)	–	9 (75.0)	7 (58.3)	*0.386*	0	0	–	3 (30)	10 (100)	***<0.001***
**Shape**	Elliptical	7 (58.3)	10 (83.3)	*0.178*	6 (60)	0	***0.003***	7 (58.3)	8 (66.7)	*0.673*	1 (14.3)	0	*0.299*	0	0	–
	Square	0	1 (8.3)	*0.307*	4 (40)	10 (100)	***0.003***	2 (16.7)	2 (16.7)	*1.0*	6 (85.7)	7 (100)	*0.299*	9 (90)	9 (90)	*0.593*
	Round	5 (41.7)	1 (8.3)	*0.059*	0	0	–	3 (25.0)	2 (16.7)	*0.615*	0	0	–	1 (10)	2 (20)	*0.593*
	Slit-like	0	0	–	0	0	–	0	0	–	0	0	–	0	0	–
**Postdilation measures**	Weft-aligned diameter (mm)	4.8 (5.2–4.4)	4.8 (4.9–4.5)	*0.483*	2.8 (3.0–2.4)	2.5 (2.6–2.3)	***0.027***	4.2 (4.5–3.7)	4.5 (4.6–4.2)	*0.250*	3.0 (3.2–2.4)	2.9 (3.0–2.7)	*0.829*	3.2 (3.7–2.7)	2.7 (3.0–2.6)	***0.022***
	Warp-aligned diameter (mm)	3.1 (3.2–3.0)	3.1 (3.2–3.1)	*0.476*	3.2 (3.3–3.0)	3.0 (3.2–2.6)	*0.050*	2.8 (3.0–2.6)	2.6 (2.7–2.4)	***0.020***	3.3 (3.7–3.0)	3.1 (3.5–2.9)	*0.554*	2.7 (3.7–2.4)	3.4 (3.7–2.8)	*0.177*
	Surface (mm^2^)	13.0 (13.8–11.9)	12.8 (13.7–12.1)	*0.385*	7.8 (8.6–6.7)	6.5 (7.0–5.7)	***0.007***	10.4 (11.7–9.2)	10.5 (11.3–10.1)	*0.599*	8.3 (8.6–7.4)	7.9 (9.1–7.4)	*0.738*	8.1 (9.8–7.8)	8.5 (9.3–8.3)	*0.251*
**24-hours assessment**	Weft-aligned recoil (%)	15.1 (25.2–9.3)	14.5 (21.4–7.9)	*0.273*	11.8 (16.4–7.7)	19.5 (24.8–6.5)	*0.078*	15.9 (25.2–11.2)	15.9 (25.1–8.2)	*0.307*	6.4 (10.5–0.8)	6.6 (10.1–0.3)	*0.444*	10.0 (18.9–5.7)	6.4 (9.2–4.3)	*0.197*
	Warp-aligned recoil (%)	14.2 (23.4–11.7)	13.3 (18.1–9.7)	*0.216*	16.4 (23.5–13.9)	11.3 (16.5–4.1)	***0.031***	17.0 (23.6–9.1)	18.2 (24.5–11.5)	*0.215*	14.7 (18.8–5.4)	6.8 (13.3–3.6)	*0.150*	9.3 (14.8–6.9)	16.7 (23.1–11.3)	***0.022***
	Surface recoil (%)	18.5 (23.8–8.9)	24.8 (28.1–14.8)	*0.074*	21.1 (23.1–19.2)	20.2 (29.7–15.1)	*1.0*	25.1 (31.8–18.8)	33.6 (38.9–25.4)	***0.026***	5.5 (8.4–0.9)	7.4 (10.1–1.3)	*0.191*	13.0 (17.7–10.3)	12.2 (19.2–2.5)	*0.127*
	Surface (mm^2^)	10.7 (11.4–9.6)	9.8 (10.8–9.0)	*0.446*	6.2 (6.6–5.4)	5.0 (5.3–4.8)	***<0.001***	7.9 (8.5–6.9)	6.8 (8.5–6.2)	*0.178*	8.0 (8.5–7.6)	7.4 (8.4–7.2)	*0.259*	7.0 (8.9–6.4)	8.1 (8.6–6.8)	*0.326*

Data presented as median (IQR). Significant *p*-values (<0.05) in bold italics. Group A = low energy; Group B = high energy.

## Data Availability

Data is contained within the article. The original contributions presented in this study are included in the article.

## Abbreviations

The following abbreviations are used in this manuscript:

BSG	Bridging Stent graft
ISF	In situ fenestration
L-ISF	Laser in situ fenestration
OTW	Over-the-wire
PBD	Plain balloon dilatation
PTA	Percutane transluminal angioplasty
TVs	Target vessels
